# The 2020 Pandemic: Current SARS-CoV-2 Vaccine Development

**DOI:** 10.3389/fimmu.2020.01880

**Published:** 2020-08-19

**Authors:** Sana O. Alturki, Sawsan O. Alturki, Jennifer Connors, Gina Cusimano, Michele A. Kutzler, Abdullah M. Izmirly, Elias K. Haddad

**Affiliations:** ^1^Department of Microbiology and Immunology, Drexel University College of Medicine, Philadelphia, PA, United States; ^2^Department of Medical Technology, Faculty of Applied Medical Sciences, King Abdulaziz University, Jeddah, Saudi Arabia; ^3^Division of Infectious Diseases and HIV Medicine, Department of Medicine, Drexel University College of Medicine, Philadelphia, PA, United States

**Keywords:** Severe Acute Respiratory Syndrome Coronavirus 2 (SARS-CoV-2), coronavirus disease 2019 (Covid-19), antibody dependent enhancement (ADE), receptor binding domain (RBD), pandemic, clinical trial, spike (S) protein

## Abstract

Coronaviruses are enveloped viruses with a positive-sense single-stranded RNA genome infecting animals and humans. Coronaviruses have been described more than 70 years ago and contain many species. Severe Acute Respiratory Syndrome (SARS) and Middle East Respiratory Syndrome (MERS) are lethal species caused by human coronaviruses (HCoVs). Currently, a novel strain of HCoVs, named Severe Acute Respiratory Syndrome Coronavirus 2 (SARS-CoV-2) causes coronavirus disease 2019 (Covid-19). SARS-CoV-2 was first identified in December 2019 in Wuhan, the capital city of the Hubei province of China, and has since spread worldwide causing an outbreak in more than 200 countries. The SARS-CoV-2 outbreak was declared a pandemic on March 11th, 2020 and a public health emergency of international concern (PHEIC) in late January 2020 by the World Health Organization (WHO). SARS-CoV-2 infects the respiratory tract causing flu-like symptoms and, in some, may cause severe illness like pneumonia and multi-organ failure leading to death. Today, Covid-19 cases almost reaching 9 million, with more than 450 thousand deaths. There is an urgent demand for developing a vaccine since no effective therapies or vaccines have been approved to this day to prevent or minimize the spread of the infection. In this review, we summarized the furthest vaccines in the clinical pipeline.

## Coronavirus Overview

Coronaviruses are a group of enveloped viruses containing a positive-sense, single-stranded RNA genome (1). Coronaviruses originate from animals such as birds, bats, and camels (1–3) and may cause mild disease such as the common cold to severe illnesses in the respiratory track when infecting humans (4). In 2002, Severe Respiratory Acute Syndrome Coronavirus (SARS-CoV) and in 2012 Middle East Respiratory Syndrome were previous outbreaks that caused a great public health threat (5, 6). In December 2019, a novel strain of coronavirus was identified after an outbreak of pneumonia was reported in Wuhan, China (7). In February 2020, this virus was named “Severe Acute Respiratory Syndrome Coronavirus 2” (SARS-COV-2) by the Coronavirus Study Group (CSG) of the International Committee (8). SARS-CoV-2 causes a potentially fatal disease which the World Health organization has named “Covid-19” in February 2020 (9). Since June 23rd 2020, more than 8.9 million cases were reported worldwide with more than 469 thousand known deaths. Across the United states alone, nearly 2.3 million confirmed cases have been reported with more than 119 thousand deaths making it the leading country in the number of Covid-19 confirmed cases and deaths followed by Brazil with more than 1 million cases and Russia with more than 599 thousand confirmed cases. Currently, the pandemic is underway and many more people are getting infected globally (10).

## Clinical Manifestations/Symptoms

**Figure 1:**
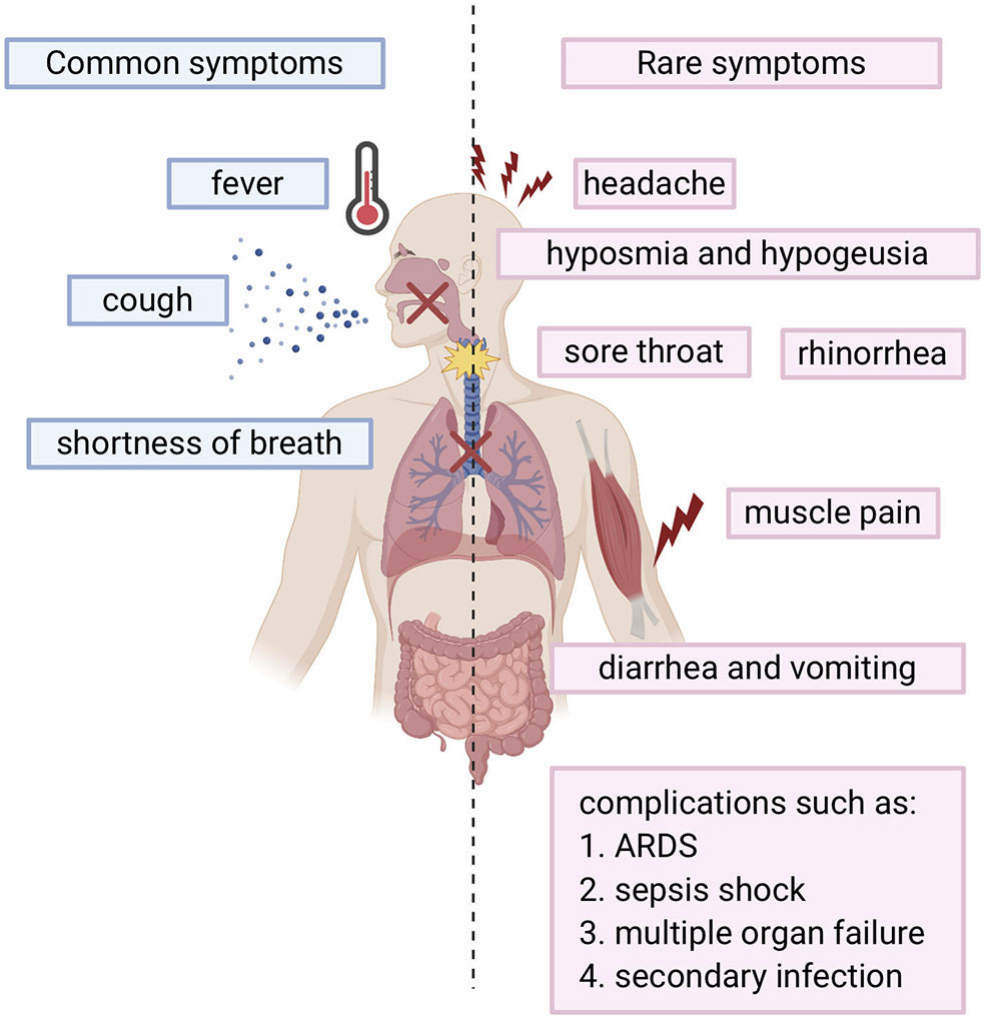
Overview of COVID-19 symptoms: Blue bars represent the common symptoms. Pink bars represent the rare symptoms.

SARS-COV-2 infected individuals experience a wide range of symptoms which differs from one person to another. The clinical manifestations range from mild to severe illness and sometimes, death. Usually, symptoms develop anywhere from 2 days to 2 weeks after being exposed to the virus (11). The most common symptoms include fever, cough, shortness of breath, and fatigue (12, 13). Other symptoms include rhinorrhea, sputum production, headache, and sore throat. In addition, some individuals may display rare symptoms like gastrointestinal symptoms including diarrhea and vomiting. Many other symptoms may also develop such as hyposmia (reduced ability to smell) and hypogeusia (reduced ability to taste) (14). In more severe cases, individuals may require hospitalization and even admission into the intensive unit care (13, 15). Disease of these patients may quickly progress and cause complications such as acute respiratory distress syndrome (ARDS), sepsis shock, multiple organ failure, and secondary infection which may eventually lead to death in a short period of time (13, 15). Risk factors that contribute to more severe illness and critical conditions include age (the elderly over 65) and general health status (those with underlying disorders) including those with hypertension, cardiovascular disease, diabetes, and those with a weakened immune system. (13). Fortunately, many individuals experience only mild symptoms or were asymptomatic (16, 17). Symptoms are summarized in Figure 1.

## Transmission

**Figure 2:**
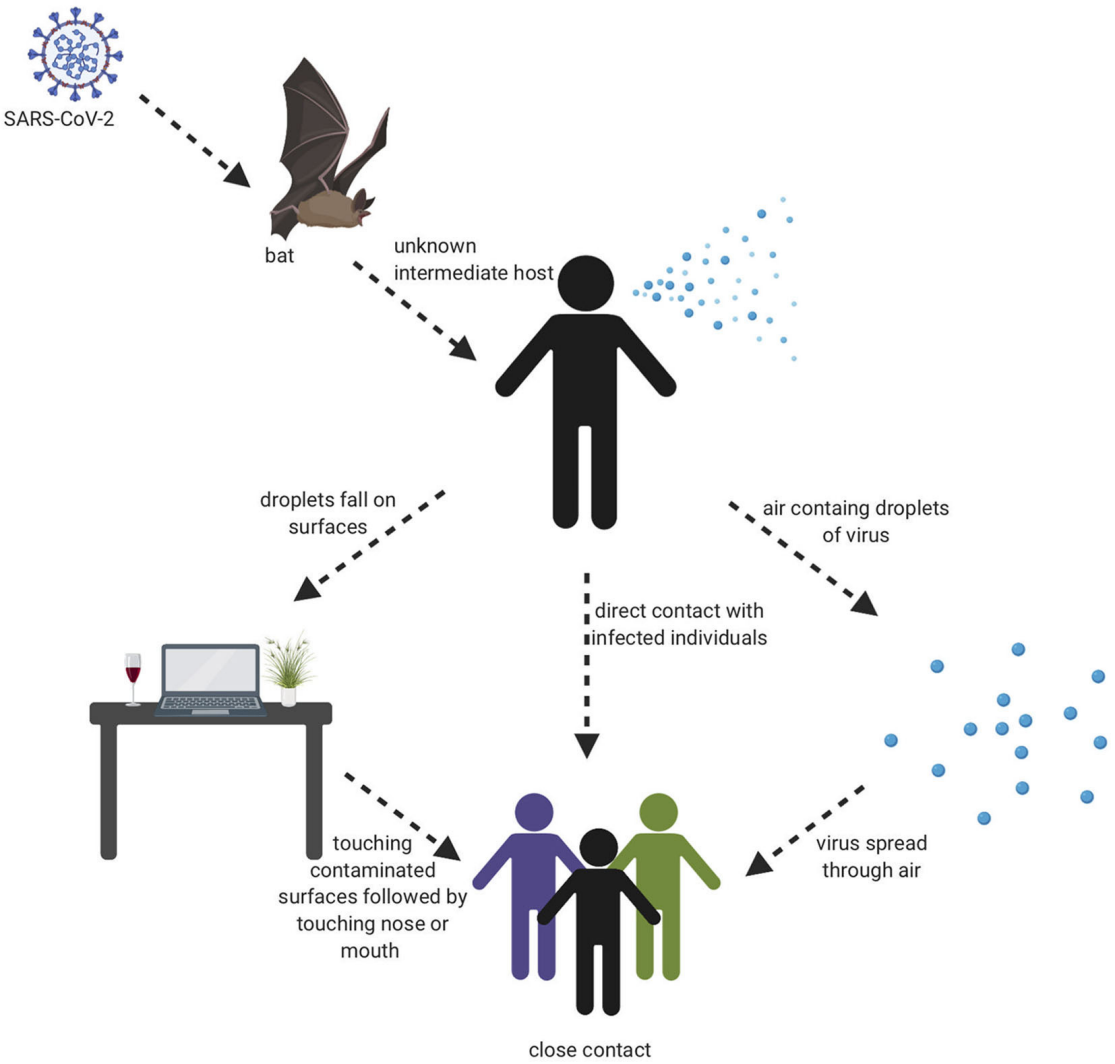
Transmission cycle of SARS-CoV-2. It is believed that animal to human transmission is the main cause of infection through consuming an infected animal as a source of food. Then, the infection can spread through person to person, aerosols, and touching contaminated surfaces.

The beginning of the SARS-COV-2 pandemic has been traced back to the animal wholesale market in Wuhan, China where many Covid-19 cases first appeared after infected individuals visited that market (7, 18). It is believed that SARS-COV-2 likely originated from animals and that animal to human transmission is the main mechanism of transmission (7, 19). When the genome sequence of SARS-COV-2 was analyzed, it showed ~90% similarity with bat SL-CoVZXC21 and bat-SL-CoVZC45 (20). This finding suggested that mammals are most likely to be the link between SARS-COV-2 and human. Other studies concluded that person-to-person may be the route of transmission since many Covid-19 cases were reported among family members and those that had been in contact with an infected person without any previous visits to the Wuhan animal market. It has shown that infected people could spread the virus even before any symptoms develop and asymptomatic individuals could spread the virus as well (21). There are many ways the virus can spread from person to person either through direct contact or through droplets when sneezing or coughing by an infected person (22). A study was done in different environmental conditions to evaluate the stability of SARS-CoV-2 in aerosols and on various surfaces. The study data showed that SARS-CoV-2 remained viable for 3 h in aerosols. On cardboard, the virus remained viable for 24 and 72 h on stainless steel and plastic. In addition, the virus viability lasted 4 h on copper (23). The severity level of the SARS-CoV-2 disease is much less compared to MERS and SARS-CoV, however, the infectiousness level of SARS-CoV-2 is much higher than other coronaviruses, which probably is due to viral shedding, incubation time, and binding strength to its receptor, angiotensin-converting enzyme 2 (ACE2). The infectiousness of SARS-CoV-2 resembles influenza more than SARS-CoV since the infectiousness reaches its highest level shortly around or even before the onset of symptom. In other words, the infected patients spread SARS-COV-2 before they have developed symptoms due to the rapid shedding of the virus which begins 2–3 days before the appearance of the first symptoms. Then, the viral load decreases significantly after 8 days of developing the onset of symptoms (24). Recently, a group identified a unique furin cleavage site at the S1/S2 boundary of SARS-CoV-2 S protein setting it apart from SARS-CoV (25). It is possible that this furin cleavage motif has contributed to the expanded tropism and transmission of SARS-CoV-2 and the high affinity binding to ACE2. It should be noted, that since individuals who are asymptomatic spread the virus, and the virus spread could cause pandemic within weeks, thus prevention precautions such as quarantine and isolation are difficult to achieve. This evidence leads to the belief that a vaccine is an extremely important goal to prevent future spread of this disease. Visual depiction of SARS-COV-2 transmission is shown in Figure 2.

## Structure and Life Cycle

SARS-CoV-2 is a single stranded RNA virus that belongs to the Betacoronavirus (betaCoVs) genus. It has a diameter of ~60–140 nm in size and a genome of 29,891 nucleotides that encodes for 9,860 amino acids. Studies have shown that SARS-CoV-2 shares ~80% of its sequence identity with SARS-CoV (26). This genomic analysis suggests that this virus may have originated and evolved from bats. The virus's genome encodes for ~16 non-structural proteins (NSP) and 4 major structural proteins. The structural proteins include the spike (S) protein, the envelope (E) protein, membrane (M) protein, and the nucleocapsid (N) protein (26). The S protein mediates the binding of SARS-CoV-2 to ACE2 on the host cell which leads to virus entry and pathogenesis. The S protein consists of two subunits (S1 and S2). S1 subunit carries the receptor binding domain (RBD) that directly interacts with the ACE2 receptor, while S2 subunit mediates the membrane fusion of the virus-host cell by containing the essential elements required for this process (26). The N protein forms the nucleocapsid and is responsible for mRNA transcription and RNA replication. It is also responsible for the virus budding through signal transduction (27). The M protein plays a role in the assembly of the virus, while the E protein is considered to be a major virulence factor and plays a role in the secretion of inflammatory factors (28, 29).

**Figure 3:**
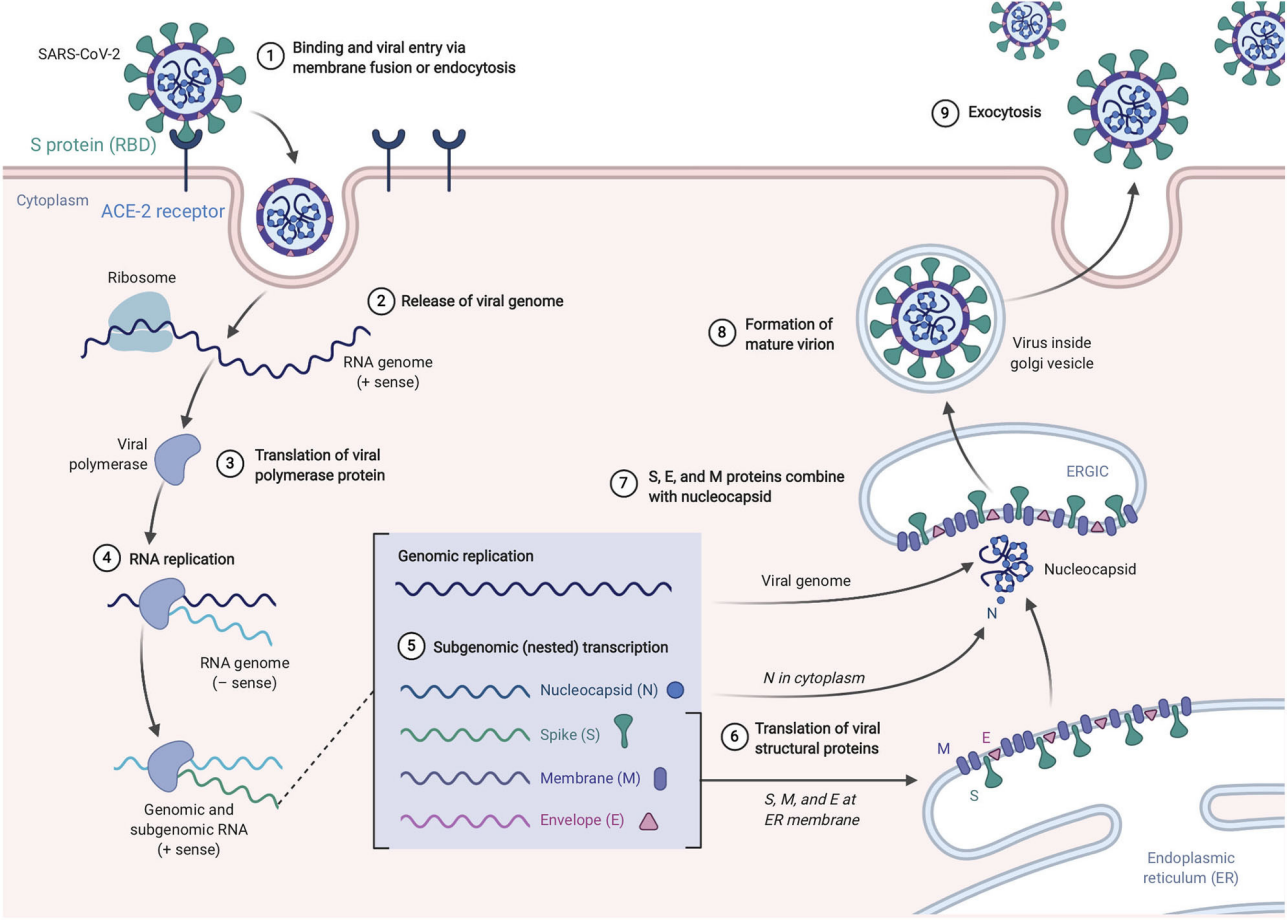
SARS-Cov-2 life cycle. The virus enters and replicates in cells that express ACE2 such as lung epithelia, myocardial, renal, GI tract, and hepatic cells. The mature virions are released from the primary host cells via exocytosis and infect new target cells. Figure from Biorender.com.

SARS-CoV-2, like its antecedent SARS-CoV, spreads through respiratory droplets and through fomite transmission (23). After exposure, the viral S proteins bind to ACE2 receptors that are broadly expressed in a variety of cells including respiratory epithelia and alveolar monocytes and macrophages as well as myocardial, renal, hepatic, and gastrointestinal tract tissues (30–32). SARS-CoV-2 may also utilize CD209L and CD147 as an alternative receptors like SARS-CoV though with much lower affinity (33, 34). The use of these alternate receptors may partially explain why the transmission rate of SARS-CoV-2 is so high as they will allow for potent infectivity even on cells expressing low ACE2. This entry mechanism depends upon cellular proteases including human airway trypsin-like protease (HAT) (35), transmembrane protease serine 2 (TMPRSS2) (36), and cathepsins. These proteases function to split the spike protein for further penetration. Following fusion of the viral envelope to the host membrane, the viral RNA is released into the cytoplasm. Open reading frame 1a (ORF1a) and ORF1b are then translated into the overlapping polyproteins, pp1a and pp1ab which are cleaved by the viral papain-like proteases and a serine type Mpro (chymotrypsin-like proteases) that are encoded by ORF1a to produce 16 non-structural proteins that form the RNA replicase-transcriptase complex (RTC). Viral RNA synthesis produces both genomic and sub-genomic RNAs, the latter which serves are mRNA for 7–9 structural proteins such as the E, N, M, and S proteins that are produced through discontinuous transcription (37). Both genomic and sub-genomic RNAs are produced through negative-sense (-RNA) intermediates via the RNA-dependent RNA polymerase (RdRp) (1). The viral nucleocapsids are then assembled with N-protein encapsidated genomic RNA in the cytoplasm. The assembled viral nucleocapsid buds into the lumen of the endoplasmic reticulum-Golgi intermediate compartment (ERGIC) and the completed, mature virion is released from the infected cell through exocytosis (38). Depiction of the life cycle is shown in Figure 3.

**Figure 4:**
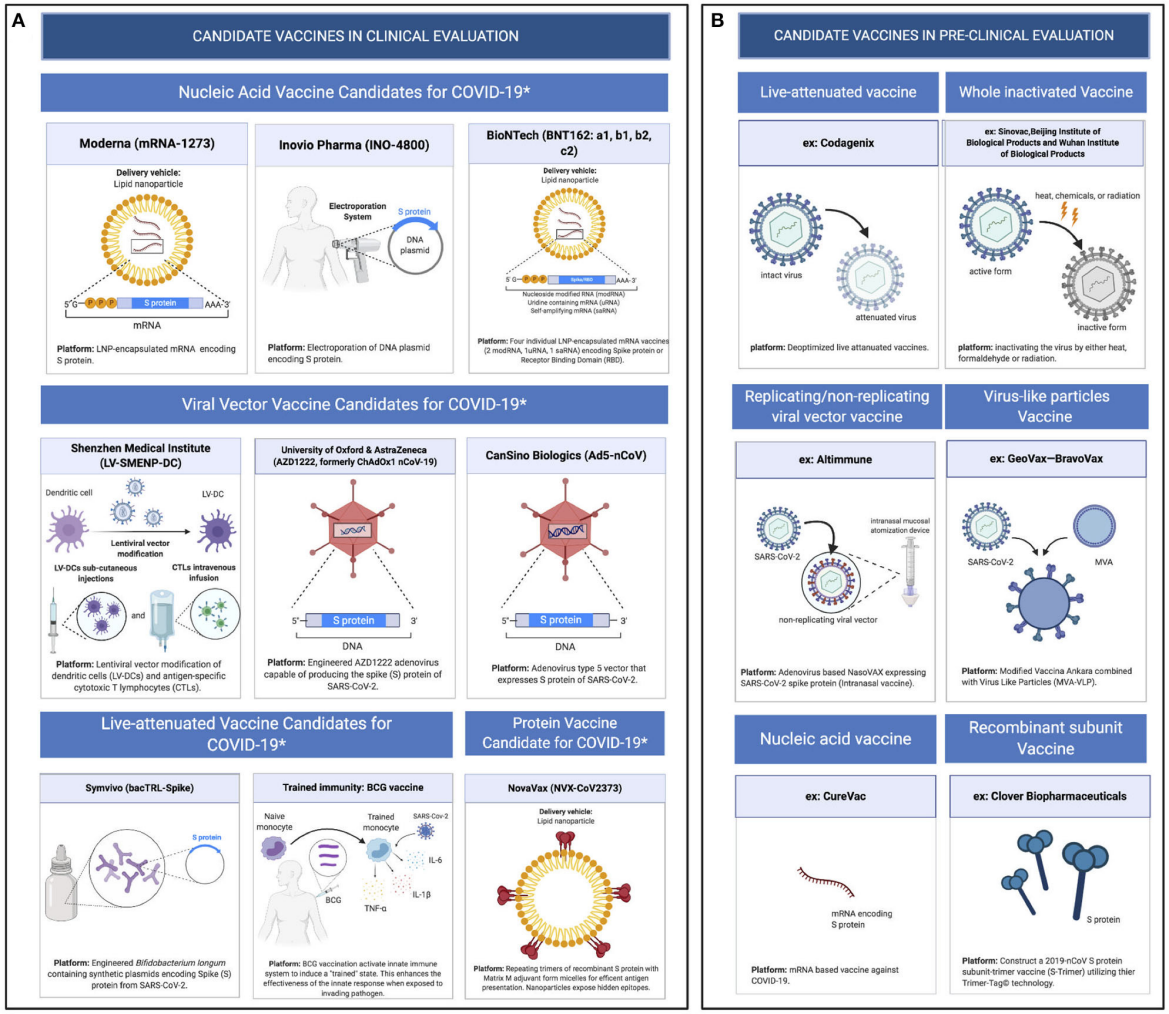
Pipeline of COVID-19 vaccine candidates' platforms. **(A)** Ongoing vaccine development platforms for SARS-CoV-2 (live attenuated, nucleic acid, viral vector, and protein vaccines). **(B)** Pre-clinical vaccine candidates' platforms (live attenuated virus, inactivated virus, replicating/non-replicating viral vector, virus like particles, subunit, and nucleic acid vaccine).

Because of the urgent need for a vaccine, WHO accelerated the vaccine development process by activating the R&D Blueprint that aims to diminish the time of research and development process, improve scientists and global health professionals coordination, and generate new norms and standards to learn from the global response and its improvement (39). Currently, more than 115 vaccine candidates are being developed. The majority are in exploratory and preclinical stages however, around ten vaccine candidates have advanced recently and moved into clinical development (Table 1). These include mRNA-1273, developed by Moderna, Ad5-nCoV, developed by CanSino Biologicals, INO-4800, developed by Inovio, LV-SMENP-DC, and pathogen-specific aAPC, developed by Shenzhen Geno-Immune Medical Institute, and AZD1222 nCoV-19 formerly known as ChAdOx1, developed by the University of Oxford (Figure 4A). Numerous other companies and vaccine developers are on their approach to initiate human testing in a couple of months including Viroclinics Xplore. Various vaccine platforms are being used, including live attenuated virus, inactivated virus, peptide-based, virus-like particles, replicating/non-replicating viral vector, recombinant protein, and nucleic acid (DNA and RNA) (Figure 4B) (40–46). Following the public release of the complete sequence of the SARS-CoV-2 genome on January 12, 2020, numerous nucleic acid-based vaccine candidates have emerged including mRNA-1273, INO-4800, and AZD1222 nCoV-19 (47). Most of nucleic acid vaccines are based on the major antigen S protein coding sequence. RBD on S1 can recognize different type of receptors (48). For instance, MERS-CoV recognizes dipeptidyl peptidase 4 (DPP4) as its receptor whereas SARS-CoV recognizes ACE2 (49). It has been reported that the sequence and the structure of SARS-CoV-2 is more like SARS-CoV than MERS-CoV however, SARS-CoV-2 S protein shares a global protein fold architecture with the MERS-CoV S protein (50). Despite the strong structural homology between the two RBDs of SARS-CoV and SARS-CoV-2, studies demonstrated a limited antibody cross reactivity between SARS-CoV RBD-specific monoclonal antibodies to 2019-nCoV S protein (51, 52). Therefore, developing a vaccine targeting RBD can inhibit viral attachment hence, fusion.

**Table 1:** List of clinical-phase vaccine candidates for COVID-19 and clinical trial status as of June 2020.

**Phase I**				
**Name**	**Developer**	**Method**	**Trial**	**Trial enrollment**
bacTRL-spike	Symvivo corporation	Orally administered probiotic bacteria encoding the SARS-COV-2 S protein	NCT04334980	Not yet recruiting
NVX-CoV2373	Novavax	Recombinant S protein made using Novavax's proprietary nanoparticle technology and includes Matrix-M adjuvant	NCT04368988	Recruiting
INO-4800	Inovio pharmaceuticals	DNA vaccine administered intradermally followed by CELLECTRA® electroporation (EP)	NCT04336410	
AZD1222 nCoV-19 formerly known as (ChAdOx1)	University of oxford	Chimpanzee adenovirus vector carrying gene for the SARS-COV-2 S protein	NCT04324606	
mRNA-1273	Moderna	Lipid nanoparticle encapsulating mRNA for SARS-COV-2 S protein	NCT04283461	
Covid-19 aAPC	Shenzhen geno-immune medical institute	Lentiviral vector expressing SARS-COV-2 proteins and immunomodulatory genes to modify artificial antigen presenting cells and active T cells	NCT04299724	
LV-SMENP DC			NCT04276896	
BNT162	Pfizer and BioNTech	Lipid nanoparticle encapsulating mRNA for SARS-COV-2 S protein	NCT04368728	
**Phase II**				
Ad5-nCoV	CanSino Biologicals	Adenovirus encoding the SARS-COV-2 S protein	NCT04341389	Active, not recruiting
**Phase III/IV**				
BCG vaccine	Ain shams university	Intradermal or intracutaneous administration of BCG vaccine to induce non-specific protective effect on SARS-COV-2	NCT04350931	Not yet recruiting
	UMC utrecht		NCT04328441	Recruiting
	murdoch children's research institute		NCT04327206	
	Texas A&M university		NCT04348370	
	Bandim health project		NCT04373291	Not yet recruiting
	Hellenic institute for the study of sepsis		NCT04414267	Recruiting
	Assistance publique–hôpitaux de paris		NCT04384549	Not yet recruiting
	Universidad de antioquia		NCT04362124	
	Radboud university		NCT04417335	Active, not recruiting
	University of Campinas, Brazil		NCT04369794	Not yet recruiting
	TASK applied science		NCT04379336	Recruiting

## Current Vaccines

### Phase I Clinical Trials

#### Symvivo

Symvivo Corporation is leading an ongoing Phase I clinical trial evaluating the safety, tolerability, and immunogenicity of their bacTRL-spike vaccine for Covid-19 prevention (NCT04334980). Once orally administered, the bacTRL-spike vaccine is designed such that the genetically modified probiotic bacteria, *Bifidobacterium longum*, should colonize the gut, bind to intestinal epithelial cells, replicate, secrete, and deliver plasmids expressing the SARS-CoV-2 spike protein. As a living-medicine, the expression of the SARS-CoV-2 S protein is expected to be sustained throughout the life of the colonized *B. longum*. This results in continued delivery and expression of SARS-CoV-2 S protein encoding plasmids. Translation of this plasmid within the gastrointestinal lymphoid tissues is expected to initiate a robust mucosal, systemic humoral, and cell-mediated immune response in treated patients. This Phase I clinical trial will enroll 84 healthy adult volunteers who will receive 1, 3, or 10 billion colony forming units (CFU) of the live, genetically modified *B. longum* alongside placebo treated control patients. Unlike typical vaccine administration, the bacTRL-spike vaccine will be administered as an oral, lyophilized gel-capsule similar to traditional consumer probiotic supplements (53). Patients will be monitored over the course of 12 months to measure outcomes including but not limited to the production, seroconversion, and stability of SARS-CoV-2 S antibodies, intestinal colonization of the genetically modified *B. longum*, and frequency of adverse events. This study is being conducted at BC Children's Hospital Research Institute and The Canadian Center for Vaccinology and is anticipated to be completed by December 31, 2021.

#### Novavax

NVX-CoV2373, a vaccine being developed by Novavax, is a recombinant S protein (SARS-CoV-rS) vaccine that is made using Novavax's proprietary nanoparticle technology and includes Novavax's Matrix-M saponin-based adjuvant. Matrix-M is a potent inducer of leukocyte migration into the draining lymph nodes (LN) resulting in the increase in T-, B-, NK, and dendritic cells in draining LNs. These recruited cells also showed an upregulation of activation markers (54, 55). This adjuvant, when compared with other adjuvants like Alum, AS03, and FCA, is a more potent alternative (56). In pre-clinical trials, NVX-CoV2373 was shown to be highly immunogenic in animal models. NVX-CoV2373 produced high levels of S protein-specific antibodies that can block ACE-2 human RBD and wild-type SARS-CoV-2 neutralizing antibodies after one dose. After a second dose, the neutralization titers jumped eight-fold (57). NVX-CoV2373 will enter clinical trials in mid-May and will undergo a Phase I/Phase II approach (NCT04368988). This study will first enroll 131 subjects to assess the dosage amounts and the number of doses required with and without Matrix-M adjuvant. The study will start with 5 or 25 μg of SARS-CoV-rS with or without equal amounts of Matrix-M. It will also look at 25 μg SARS-CoV-rS with 5μg of Matrix-M. Each arm of this study will receive two intramuscular (IM) injections at a day 0 and day 21. Primary outcome measures will include looking at frequency of adverse events and serum IgG antibody levels specific for SARS-CoV-2 rS protein.

#### Inovio

INO-4800 is a synthetic DNA-based vaccine developed by Inovio pharmaceuticals collaborating with researchers at the Wistar Institute, Seoul National University Hospital, and the International Vaccine Institute (IVI). INO-4800 vaccine is a synthetic DNA-based vaccine that are delivered into human cells via electroporation (EP) and translated into S proteins to induce an immune response. The main advantage of the nucleic acid-based platform is an accelerated developmental timeline due to their ability to be designed rapidly, manufactured in large quantities, and have great flexibility in terms of antigen manipulation (58, 59). In pre-clinical trials, immunizing animal models with INO-4800 has shown to rapidly provoke the stimulation of T cells and potent RBD binding antibodies following a single immunization (60). Muthumani et al. have extensive experience with DNA vaccine against coronavirus as they have demonstrated the ability of a DNA vaccine encoding the MERS S protein (INO-4700) to induce a potent cellular immunity, antigen-specific neutralizing antibodies, and provide protection in NHP challenge models (61). INO-4800 utilizes a strategy identical to the DNA vaccine for MERS INO-4700. Currently, INO-4700 is in clinical testing in week 16 from a Phase 1/2a trial in South Korea. INO-4700 clinical tests outcome showed very promising results in which 92% of the vaccine recipients displayed the ability to neutralize the virus and 84% of vaccine recipients, after the third dose of 0.6 mg of INO-4700, showed robust T cell responses. From the previous studies, INO-4800 shows a promising vaccine candidate against SARS-CoV-2. DNA vaccines are safe since they are unable to revert into active forms. Besides offering long-term stability for ease of storage and transport (cold chain free), DNA vaccines induce strong cellular and humoral responses making the DNA vaccine an ideal approach. Inovio started testing *in-vivo* and *in-vitro* expression and immunogenicity of INO-4800 just 6 weeks after SARS-CoV-2 genome sequence identification. Currently, it has entered a phase 1 clinical trial (NCT04336410) to evaluate its safety, tolerability, and immunological profile. It will be administered intradermally (ID) on day 0 and week 4 of 1.0 mg per dosing visit followed by electroporation (EP) using the CELLECTRA^®^ 2,000 device in healthy adult volunteers (60).

#### University of Oxford

AZD1222, formally ChAdOx1, nCoV-19 vaccine, developed in the UK by Jenner Institute of University of Oxford, contains the genetic sequence of the SARS-COV-2 S protein with a transgenic, non-replicating chimpanzee adenovirus-based vector (62). This viral vectored vaccine platform has a great advantage since it leads the host cells to express the coronavirus S protein thus leading to the stimulation and production of a robust humoral and T cell-mediated immune response upon immunization (63). This platform is currently being used for a MERS vaccine and has completed phase I clinical trial (NCT03399578) (64, 65). The SARS-COV-2 vaccine trial is now recruiting for phase I/II combined clinical trial (NCT04324606) (62, 66). The non-replicating feature of this vaccine makes it relatively safe in individuals with underlying diseases and children (67). In this trial, a total of 1112 healthy volunteers aged 18–55 years will be enrolled. In this study, viral particles (vp) of AZD1222 nCoV-19 vaccine will be delivered to the experimental groups with the Meningococcal conjugate vaccine (MenACWY) used as a control. Volunteers will be divided into 4 groups (Groups: 1a, 1b, 2a, 2b, 3, 4a, 4b, 4c, 4d). Experimental groups 1a, 2a, 4a will receive a single dose of 5 × 10^10^vp AZD1222 nCoV-19 while group 3 will receive one dose of 5 × 10^10^vp AZD1222 nCoV-19 at week 0 and one dose of 2.5 × 10^10^vp AZD1222 nCoV-19 at week 4. Group 4c will receive a single dose of 5 × 10^10^vp AZD1222 nCoV-19 plus Paracetamol. In addition, active comparator groups 1b, 2b, 4b, and 4d will receive a standard single dose of MenACWY (IM) plus Paracetamol for group 4d. Paracetamol is an antipyretic/analgesic drug known to reduce fever and pain (febrile reactions) and is generally used after vaccination. However, in this trial the main purpose of using this drug was to assess safety, reactogenicity, immunogenicity, and efficacy for participants receiving prophylactic Paracetamol. It is important to include this arm of the study because it has been shown that prophylactic Paracetamol administration at the time of vaccination or during the first 6–8 h post vaccination could impact the immune response negatively to several vaccine antigens in children and adults (66, 68–70). The time frame of this study will be ~6 months with a follow up visit at Day 364 (66). Primary outcome measures will be to assess the efficacy and adverse events of AZD1222 nCoV-19. Secondary outcome measures will be to assess the safety, tolerability, and reactogenicity profiles of the candidates (66).

#### Moderna

mRNA-1273 is created by Moderna Inc in collaboration with National Institute of Allergy and Infectious Diseases (NIAID) and Coalition for Epidemic Preparedness Innovations (CEPI). It is similar to INO-4800 where both vaccines encode the S protein of SARS-CoV-2 that is translated by host cells following vaccination and will mimic a natural infection immune response. In this study, the developers used a novel mRNA encapsulated within lipid nanoparticles (LNPs) composed of ionizable lipid, distearoyl phosphatidylcholine, cholesterol, and polyethylene glycol lipid. Vaccination by mRNA/LNP is a novel approach that has improved its ability to elicit strong immune responses in numerous pre-clinical and clinical studies developed by Moderna including CMV, Zika virus, H7N9, hMPV, and RSV (71, 72). Formulation of the mRNA antigen within an LNP improves immunogenicity, protecting the mRNA from enzymatic degradation, and facilitating efficient uptake by target cells. Moderna started its clinical trial just 42 days after the complete viral sequence has been available (NCT04283461). The study is recruiting 155 healthy males and non-pregnant females, starting at 18–99 years of age. In the primary objective, individuals will be enrolled into one of thirteen cohorts (10, 25, 50, 100, 250 μg) and will receive the vaccine IM on days 1 and 29 of 0.5 milliliter of mRNA-1273 to assess the vaccine safety and reactogenicity. If the vaccine passes the primary objective and meets all safety and reactogenicity criteria, it will undergo the secondary objective to evaluate the immunogenicity. The outcome measures for this trial centers around the frequency of adverse events and determines the antibody response and percentage of subjects who have seroconverted. In May, Moderna announced positive interim phase one data for mRNA-1273. All participants among the groups who received 25 and 100 μg dose cohorts seroconverted with binding antibody levels which reached or exceeded what is seen in convalescent sera following the second dose of vaccination. Eight of these seroconverted volunteers had neutralizing antibody titers at or beyond what is generally seen in convalescent sera. In addition, viral challenge studies in mice showed complete protection against viral replication in their lungs after vaccination with mRNA-1273. This immunogenicity increased in a dose-dependent manner that was seen in all 45 participants at the three dose levels, and it was primed and boosted within the 25 and 100 μg dose levels. The results showed that mRNA-1273 is generally safe and well-tolerated. However, a few of the participants experienced grade 3 adverse events such as erythema around the injection site which were transient and self-resolving. No grade 4 or serious adverse events have been noticed. The age of the participants should be considered for the positive results due to the fact that usually younger individuals tend to show better response to the vaccine than if they were mostly from the senior group who are at a higher risk. The potential advantages of an mRNA approach include the ability to mimic natural infection, stimulate a potent immune response by combining multiple mRNAs into a single vaccine, and rapid discovery and design for a quicker respond to emerging pandemic threats (73, 74). Recently, on May 7th 2020, Moderna received an approval from the FDA to begin phase two studies which are expected to begin soon.

#### Shenzhen Geno-Immune Medical Institute

Antigen-specific T cells have a crucial role in a variety of diseases including viral infection. Generating large quantities of viral specific T cells in a rapid manner may eradicate the invasion of SARS-CoV-2. One effective method to generate massive amounts of T cells is through developing a genetically modified, artificial antigen presenting cell (aAPC) expressing SARS-COV-2-specific antigens. This is accomplished by using lentivirus vectors (LV) expressing SARS-COV-2 minigene (SMENP) and immune modulatory genes. LV are used to provoke the naïve T cells leading to effector T cell differentiation and proliferation. Dendritic cells are the ideal LV vaccine targets as they are the most potent antigen presentation cells (APCs) in which they are able to stimulate robust and durable antigen-specific T cell responses (75). LV-SMENP-DC and pathogen-specific Artificial Antigen-Presenting Cells (aAPC) are two vaccines developed by Shenzhen Geno-Immune Medical Institute, China which are based on APCs ability to stimulate viral antigen-specific T cells. Both vaccines are currently in Phase l clinical trial to evaluate their safety and reactogenicity. One hundred healthy volunteers and SARS-COV-2 infected patients will be receiving 5 × 10^6^ cells of LV-DC alone via ID injection or 5 × 10^6^ cells of LV-DC vaccine and 1 × 10^8^ antigen-specific cytotoxic T lymphocytes (CTLs) via ID injection and IV infusion, respectively (NCT04299724, NCT04276896) (76, 77). The primary and secondary outcome measures for these trials focus on the frequency of adverse events, mortality rates, and subjects with positive T cell responses.

#### Pfizer and BioNTech

BioNTech and Pfizer have started their first clinical trial for SARS-COV-2 vaccine candidate, BNT162, in Germany on April 23rd 2020 (NCT04368728). Phase I/II will enroll 200 healthy participants aged 18–55 and will receive a dose range of 1 to 100 μg. On May 5th 2020, Phase I/II clinical trial for the BNT162 vaccine program has been started in the U.S. In Phase I/II trial, 360 healthy subjects in U.S. will enroll and will be divided into two age cohorts (18–55 and 65–85 years of age). In addition, the company has partnered with Fosun Pharma to begin clinical trials of SARS-COV-2 vaccine BNT162 in China. Like mRNA-1273 from Moderna, BNT162 is a lipid nanoparticle encapsulating mRNA encoding for SARS-CoV-2 antigens. The company's development program involves four vaccine candidates, each of them demonstrating a different combination of mRNA format and target antigen (78). The primary outcome measures for this study include reports of adverse events while the secondary outcome measures focus neutralizing antibody levels.

### Phase II Clinical Trial

#### CanSino Biologics

In conjunction with CanSino Biologics, the Beijing Institute of Biotechnology (BiB) and the Jiangsu and Hubei province Centers for Disease Control and Prevention have developed a recombinant adenovirus Type 5 Vector, Ad5-nCoV, SARS-CoV-2 vaccine candidate. Ad5-nCoV contains a replication-defective adenovirus type 5 as a vector to express the full-length SARS-CoV-2 S protein (79). After successfully moving through Phase I clinical trial, Ad5-nCoV has become the first SARS-CoV-2 vaccine to move into Phase II (NCT04341389). As of review publication, CanSino Biologics have found that this vaccine in phase I trial was tolerable and immunogenic at 28 days post-vaccination with humoral responses against SARS-CoV-2 peaking at day 28 in healthy adults. Cellular responses were noted from day 14 post-vaccination though it is not yet known whether the T or B cell responses are protective (80). During phase 2,500 subjects will be enrolled, 250 subjects in the middle-dose vaccine group (5 × 1011 vp) and 125 subjects in the low-dose (1 × 1011 vp) and placebo groups, respectively. Immunogenicity will be tested on days 0, 14, 28, and 6 months post-vaccination. The vaccine will be administered through IM injections at day 0. The primary outcome measure is the occurrence of adverse events, antibody responses, specifically neutralizing antibody levels 0–14 days post-vaccination. Secondary outcome measures also include the occurrence of adverse events and antibody levels with a longer time frame (0–28 days post-vaccination). Using Ad5 as a vector comes with a potential issue known as the Ad5 set-back. Because adenoviruses cause the common cold and the average person has statistically been infected before, the presence of pre-existing immunity from natural exposure to Ad5 can dampen cellular immune responses to whatever antigens are encoded by the vector vaccine. Other Ad5 vaccines have tried to overcome this set-back using potent prime-boost regimens though the Ad5-nCoV does not appear to take advantage of this technique to overcome this potential pre-existing Ad5 immunity (81). CanSino Biologics does note in their phase one results that there is a negative effect on the pattern of T cell responses due to Ad5 pre-exiting immunity.

### Phase III Clinical Trial

#### BCG Vaccines

There are several ongoing Phase III (NCT04327206) or IV (NCT04348370) clinical trials evaluating the ability of Bacillus Calmette-Guérin (BCG) vaccines to prophylactically protect health care workers, healthy adults, and elderly populations against Covid-19. BCG vaccines are being considered to reduce the impact of Covid-19 due to its ability to induce trained immunity. Trained immunity involves the induction of metabolic and epigenetic modifications that promote an innate immune response against subsequent infections. Through trained immunity, BCG vaccines have been shown to prevent respiratory infections such as pneumonia and influenza in children and the elderly (82, 83). BCG vaccines have also been shown to reduce the severity of other viral infections. This was demonstrated in a human yellow fever infection model where BCG vaccination reduced viremia and in mouse studies where BCG vaccination reduced the severity of mengovirus infection (84–86). This non-specific protection induced by BCG vaccines may act as a stopgap while SARS-COV-2 specific vaccines are being developed (82). Patients included in these trials will receive BCG vaccines containing either the TICE, Danish 1331, or Moscow 361-1 strains of live attenuated *Mycobacterium bovis*. Patients enrolled will receive either 0.1 mL of the BCG vaccine, 0.1 mL of a saline solution as a placebo, or no immunization. BCG vaccine dosages will include 2 × 10^5^−8 × 10^5^ CFUs, 2 × 10^5^ CFUs, or 1 × 10^5^−33 × 10^5^ CFUs of *M. bovis* depending on the strain of being used. Vaccine administration will either be intradermal or intracutaneous depending on the trial. The primary outcome across most trials is Covid-19 incidence as measured by detecting SARS-CoV-2 by PCR or detection of SARS-CoV-2 Spike protein antibodies and hospital admittance. Several secondary outcomes being evaluated include but are not limited to Covid-19 symptom duration, disease severity and deaths. Study enrollment estimates vary with the lowest including 500 patients and highest including 10,078 patients. In addition to enrollment variation, study completion dates vary with the soonest estimated completion date of December 1, 2020 and latest of May 2022.

## Antibody Dependent Enhancement (ADE): A Potential Hurdle for Coronavirus Vaccine Development

Due to the presence of different strains of coronavirus and the strong structural homology between the two RBD of SARS-CoV and SARS-CoV-2, the cross-reactivity between antibodies of different coronaviruses must be taken into consideration for SARS-CoV-2 vaccine development. Antibodies have a dual role in controlling infections in which either they neutralize the infection or enhance pathogen uptake (87). Several viruses rely on pre-existing antiviral antibodies for their entry into the target cells, a mechanism known as antibody-dependent enhancement (ADE). Pre-existing antiviral antibodies from heterologous strains can prevent the virus entry to the cells by blocking the binding to its natural receptor on the host cell surface. However, these antibodies could facilitate the entry of the virus to host cells through either interaction of the antibody-virus complex with FcR receptors on various immune cells or complement receptors by activating the complement classical pathway (88). Both mechanisms tend to be linked to disease exacerbation. Generally, ADE educes sustained inflammation, lymphopenia, and potentially, cytokine storm, causing severe illness, or death. Furthermore, ADE has been observed in a variety of viruses including flaviviruses, HIV, and Ebola virus (89, 90). Importantly, ADE has been extensively studied in dengue viral infections since ADE has been linked to the severity of dengue shock syndrome (91). It should be noted that ADE was linked to some vaccines, as this was demonstrated in the efficacy trials of the tetravalent dengue vaccine (CYD-TDV). In the CYD-TDV trial, they found that seronegative individuals who received CYD-TDV suffered severe dengue disease that mimics the natural secondary infection unlike seropositive individuals who had been exposed to dengue before vaccination (92).

Recently, a study demonstrated that ADE occurs not only through the typical mechanism of the presence of sub-neutralizing antibodies but also that neutralizing antibodies against RBD might be involved in ADE. This mechanism depends on the affinity, the amount, and the specificity of the antibodies (93). Furthermore, from a different group, the cross reactivity of anti-RBD polyclonal antibodies specific for SARS-CoV with RBD protein of SARS-CoV-2 pseudovirus was demonstrated (50). Moreover, ADE phenomena have been identified in SARS-CoV infections, and now potentially COVID-19. It could be hypothesized that ADE has a role in the high mortality rate in China (94).

The high mortality rate in some countries over the others could be due to prior exposure of one or more mild strains of similar coronaviruses. The data obtained from patients of Hubei region showed lymphopenia and sustained inflammation in most of the severe and death cases (95). Based on the previous information, individuals suffering the most severe disease of COVID-19 may experience the effects of antibody dependent enhancement (ADE). ADE as a complication of COVID19 should be at the forefront while developing SARS-COV-2 vaccines to avoid similar mistakes in other vaccine development like the dengue vaccine (87, 92).

## Concluding Remarks

In this review, we summarized the background and pathogenesis of SARS-CoV-2 and the front-runners in the race of SARS-COV-2 vaccine development. Currently, we are living in unprecedented times with a rapidly evolving situation and uncovering new insights about the virus every day. Due to this situation, an accelerated track for vaccine development has been put in motion. However, it is important to not jeopardize the safety of the vaccines produced and to not undermine the ADE risk factor that is known to be associated with coronaviruses (96, 97). There are many unanswered questions that need to be addressed regarding SARS-COV-2 including how the presence of antibodies will impact the clinical course and severity of the disease. We also need to determine if infection will protect you from future infection and if so, how long protection will last and what the correlates of that protection entail. We suggest harnessing the ability of a certain T helper cell subset called T follicular helper (Tfh) cells that are essential for high affinity antibodies and class switching (87). Taking what is known about ADE and coronaviruses, researchers should proceed with extreme caution and keep ADE into consideration while we move forward in SARS-COV-2 vaccine development.

## Author Contributions

SawA, JC, and GC contributed to writing sections of the paper and figure design. EH, AI, MK, and SanA contributed to writing up the paper. All authors contributed to the article and approved the submitted version.

## Conflict of Interest

The authors declare that the research was conducted in the absence of any commercial or financial relationships that could be construed as a potential conflict of interest.
